# An integrative analysis revealing POLD2 as a tumor suppressive immune protein and prognostic biomarker in pan-cancer

**DOI:** 10.3389/fgene.2022.877468

**Published:** 2022-08-23

**Authors:** Fengyun Cong, Junxian Long, Jun Liu, Zhixiang Deng, Binli Yan, Cao Liang, Xiaoliang Huang, Jinxin Liu, Weizhong Tang

**Affiliations:** ^1^ Division of Colorectal and Anal Surgery, Department of Gastrointestinal Surgery, Guangxi Medical University Cancer Hospital, Nanning, China; ^2^ Department of Gastroenteroanal Surgery, The First People’s Hospital of Nanning, Nanning, China

**Keywords:** pan-cancer, POLD2, immune checkpoints, immune infiltration, prognosis

## Abstract

**Introduction:** POLD2 is an indispensable subunit of DNA polymerase δ, which is responsible for the synthesis of the backward accompanying strand in eukaryotic organisms. Current studies have found an association between POLD2 and the development of a variety of cancers. However, its value in cancer immunotherapy has not been fully established.

**Methods:** POLD2 expression was analyzed using RNA expression and clinical data from TCGA and GTEx databases. The prognostic impact of POLD2 on tumor patients was analyzed using clinical survival data from TCGA. Gene enrichment analysis was performed using the R package “cluster analyzer” to explore the role of POLD2. We used the TIMER2 database to analyze the relationship between immune cell infiltration and POLD2 expression in TCGA. We downloaded relevant data from TCGA and analyzed the relationship between POLD2 and immune checkpoints, immunosuppressive genes, immune activating genes, chemokines and chemokine receptors.

**Results:** POLD2 was significantly overexpressed in most tumors compared to normal tissue. High POLD2 expression was significantly associated with advanced tumor stage, significantly shorter overall survival and progression-free survival. Also, we found that POLD2 expression correlated strongly with immunomodulatory genes, and significantly negatively with most immune checkpoints (PD-L1, CTLA4, TIM3, and CD28). Pathway enrichment analysis suggests that low expression of POLD2 promotes immune regulation-related pathways and high expression promotes metabolic and DNA repair-related pathways. Furthermore, tumor microenvironment analysis suggests that high POLD2 expression inhibits infiltration of CD8^+^ T cells and CD4^+^ memory T cells.

**Discussion:** In conclusion, POLD2 may be a molecular biomarker for pan-cancer prognosis and immunotherapy. It may serve as a potential target for new insights in human tumor prognosis prediction and immunotherapy assessment.

## Introduction

POLD2 (DNA Polymerase Delta 2) is a subunit of the DNA polymerase delta (DNA Pol δ) complex, the major replication enzyme in eukaryotic chromosomal DNA replication, involved in DNA replication and many forms of DNA damage repair processes. POLD2 interacts with the other three subunits to form a scaffold for the assembly of DNA Pol δ, maintaining the stability and function of the complex. (Layer et al., 2020). Meanwhile, POLD2 has a role in maintaining DNA polymerase δ function that other subunits do not possess (Brocas et al., 2010). In mammalian cells, the POLD2 protein interacts with several proteins regulating DNA metabolism and exerts its functions, including PIAS2, P21, and PDIP1 (polymeraseδ-interaction protein 1), PDIP38, PDIP46, and WRN (Warner protein) (Zheng et al., 2012). Furthermore, relevant studies confirm that POLD2 is a novel component of the DRR-TGS pathway, is involved in the maintenance of genomic integrity, and is required for the correct establishment of epigenetic markers to regulate gene expression during DNA replication (Zhang et al., 2016).

POLD2 may be the promoter of chromosomal translocations. When chromosomal translocations occur, normal proto-oncogenes are transformed into oncogenes that cause malignant transformation of cells, that is why we believe that POLD2 has the potential to act as a cancer marker. High expression of the POLD2 gene is associated with low overall survival in patients with uroepithelial carcinoma of the bladder and may affect the overall survival of patients by inhibiting the pro-apoptotic effects of chemotherapy (Givechian et al., 2018). In human glioma specimens, POLD2 showed high expression. Further studies revealed that high expression of POLD2 accelerated the proliferation of GBM cells, leading to increased invasion of A172 cells and was associated with poor patient survival (Xu et al., 2020). Other studies have also demonstrated that POLD2 is highly expressed in a variety of cancers and is also associated with poor disease prognosis. Obviously, POLD2 can provide some new ideas for cancer treatment, but we have not been well explored for the specific relationship between high POLD2 expression and carcinogenesis, therefore, further study of the mechanism of POLD2 oncogenic effect has some significance for cancer treatment.

In addition, we have found that POLD2 plays an important role in immunity. In some patients, POLD2 has a homozygous missense mutation, which affects the stability and enzymatic activity of DNA polymerase δ complex (Conde et al., 2019) (Sánchez-Ramón et al., 2019). We learned that the loss of polymerase function resulting in replicative stress-related immunodeficiency can be an etiology of primary immunodeficiency disease. A large part of the tumor susceptibility in patients with primary immunodeficiency disease arises from the same molecular defects as the immunodeficiency itself (Hauck et al., 2018). Due to the lack of tumor immune surveillance, primary immunodeficiency predisposes to different types of cancer such as lymphoma and skin cancer (Kebudi et al., 2019), but no general mechanism of tumor susceptibility in patients with primary immunodeficiency disease has been identified. Previous studies have not provided a detailed description of the POLD2 in the immune system oncogenic role in detail, so future studies should be conducted in the context of immunity.

This article focuses on the potential of POLD2 in tumor immunotherapy. The article investigated the expression levels of POLD2 in different cancers and examined the potential impact of POLD2 in the tumor microenvironment. In addition, the article investigates the correlation of POLD2 with immunomodulatory genes and immune marker sets, such as tumor mutation burden (TMB) and microsatellite instability (MSI). Overall, this work provides evidence to elucidate the immunotherapeutic role of POLD2 in cancer and contributes to further functional experimental studies of cancer therapy.

## Methods

### Data collection

We downloaded RNA expression and clinical data for The Cancer Genome Atlas (TCGA) and Genotype Tissue Expression (GTEx) from the UCSC-XENAdatabase (Goldman et al., 2020). These data allowed us to analyze the expression of POLD2 in tumor tissues of different cancer types and in adjacent normal tissues for more than 1,000 tumor samples from 33 human cancers. We also downloaded DNA copy number and methylation data from the cBioPortal database (https://www.cbioportal.org/) and ensured that the acquisition and application methods were consistent with relevant guidelines and regulations. To verify the prediction value of POLD2 in immunotherapy response, gene expression data and clinical data of GSE78220, IMvigor210, and GSE67501, which are the immunotherapy cohort of metastatic melanoma, uroepithelial carcinoma and renal cell carcinoma, were downloaded from the Gene Expression Omnibus (GEO) database.

### Expression of POLD2 associated with immune cells analysis

In the TCGA project, the TIMER2 database was used to assess the correlation between POLD2 expression and tumor immune infiltration (Li et al., 2017). We used the ESTIMATE algorithm to assess POLD2 expression in the tumor microenvironment (TME). The extent of immune cell infiltration was compared by ImmuneScore, StromalScore and ESTIMATEScore. We analyzed MSI and TMB using POLD2 expression in TCGA tumor tissue and GTEx normal tissue, respectively.

### Gene set enrichment analyses

Correlation analysis of POLD2 was performed for all genes using TCGA data. We calculated the correlation coefficients and selected the genes associated with POLD2 for gene set enrichment analysis (GSEA). GSEA was performed using the R package “clusterProfiler” to obtain gene KEGG pathway analysis and HALLMARK analysis (Subramanian et al., 2007).

### Immunomodulation analysis

We used TCGA data to analyze the correlation between POLD2 and immune regulation. The correlation between POLD2 expression and immune activation genes, immune suppression genes, chemokines and chemokine receptors was presented by heat map. *p* < 0.05 was a significant statistical result.

### Prognostic analysis

Univariate Cox regression analysis and Kaplan-Meier mapper (Goel et al., 2010) allows to assess the effect of genes on the prognosis of different cancers. We used Kaplan-Meier and Univariate Cox regression to analyze the correlation between overall survival (OS), disease-specific survival (DSS) and progression-free interval (PFI) of POLD2 in TCGA.

### Statistical analysis

We will present the data in the form of mean ± standard error (SD). POLD2 mRNA expression values from different datasets were used to create low and high POLD2 expression groups based on *p* values determined by t-tests. Kaplan-Meier survival curves were used to assess the correlation between POLD2 and prognosis. Spearman correlation test was used to assess the correlation between POLD2 expression and targets of study, including tumor mutation burden (TMB) and microsatellite instability (MSI). All statistical *p* values were two-sided with *p* < 0.05 denoting statistical significance.

## Results

### The relationship between the expression level of POLD2 in human cancer and tumor stage

**FIGURE 1:**
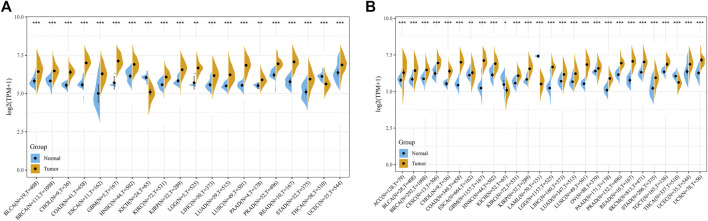
Comparison of POLD2 expression in cancer and non-cancer samples. **(A)** Expression of POLD2 in tumor and adjacent normal tissues in the pan-cancer data of TCGA cohort. **(B)** POLD2 expression in tumor tissues of TCGA and normal tissues of TCGA and GTEx cohorts. Data are shown as mean ± SD. *p < 0.05, **p < 0.01, and ***p < 0.001.

**FIGURE 2:**
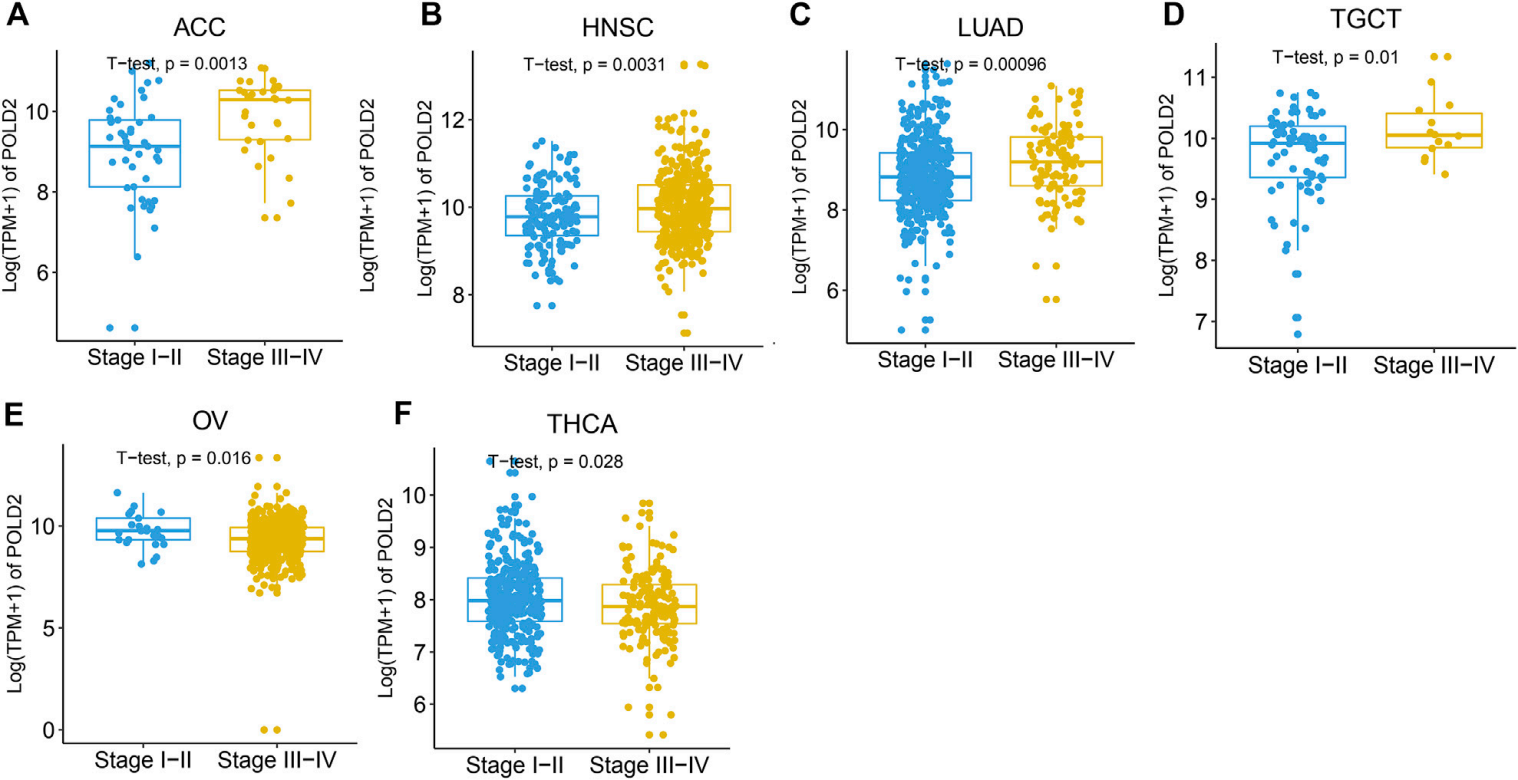
Expression levels of POLD2 in different cancer stages. **(A–F)** POLD2 expression in the different stages of cancers ACC, HNSC, LUAD, TGCT, OV, and THCA.

We evaluate the expression of POLD2 in a variety of cancers and normal samples to study the relationship between POLD2 and cancer. We first compared the expression of POLD2 in cancer and paraneoplastic tissues based on TCGA databases (Figure 1A). The results showed that the expression of POLD2 was significantly increased in BLCA, BRCA, CHOL, COAD, ESCA, GBM, HNSC, KIRC, KIRP, LGG, LIHC, LUAD, LUSC, PAAD, PRAD, READ, STAD, and UCEC (*p* < 0.05). Considering the small number of paracancerous tissue samples in the TCGA database, we included normal samples from the GTEx database for comparative analysis. The results showed that the expression of POLD2 was significantly increased in ACC, BLCA, BRCA, CESC, CHOL, COAD, ESCA, GBM, HNSC, KIRC, KIRP, LGG, LIHC, LUAD, LUSC, OV, PAAD, PRAD, READ, SKCM, STAD, TGCT, UCEC, and UCS (*p* < 0.05, Figure 1B). This indicates that POLD2 may play a facilitating role in tumor development in a variety of cancers. Then, we explored the relationship between POLD2 expression and tumor stage. It was found that as the cancer stage increased, the expression level of POLD2 in ACC, HNSC, LUAD, and TGCT increased (*p* < 0.05, Figures 2A–D). In contrast, lower POLD2 expression levels were observed only in OV and THCA at higher stages (*p* < 0.05, Figures 2E,F). The results indicate that the high level of POLD2 expression may be closely related to the advanced stage of the tumor. We performed structural analysis of this protein (Supplementary Figure S1). At the same time, we also performed functional analysis of this protein, and found that POLD2 is a component of DNA polymerase δ complex and DNA polymerase Zeta complex. POLD2 can be used as a component of trimer and tetramer DNA polymerase δ complex. POLD2 plays a role in high-fidelity genomic replication, including in lag chain synthesis and repair.

### The prognostic significance of POLD2 in human cancer

**FIGURE 3:**
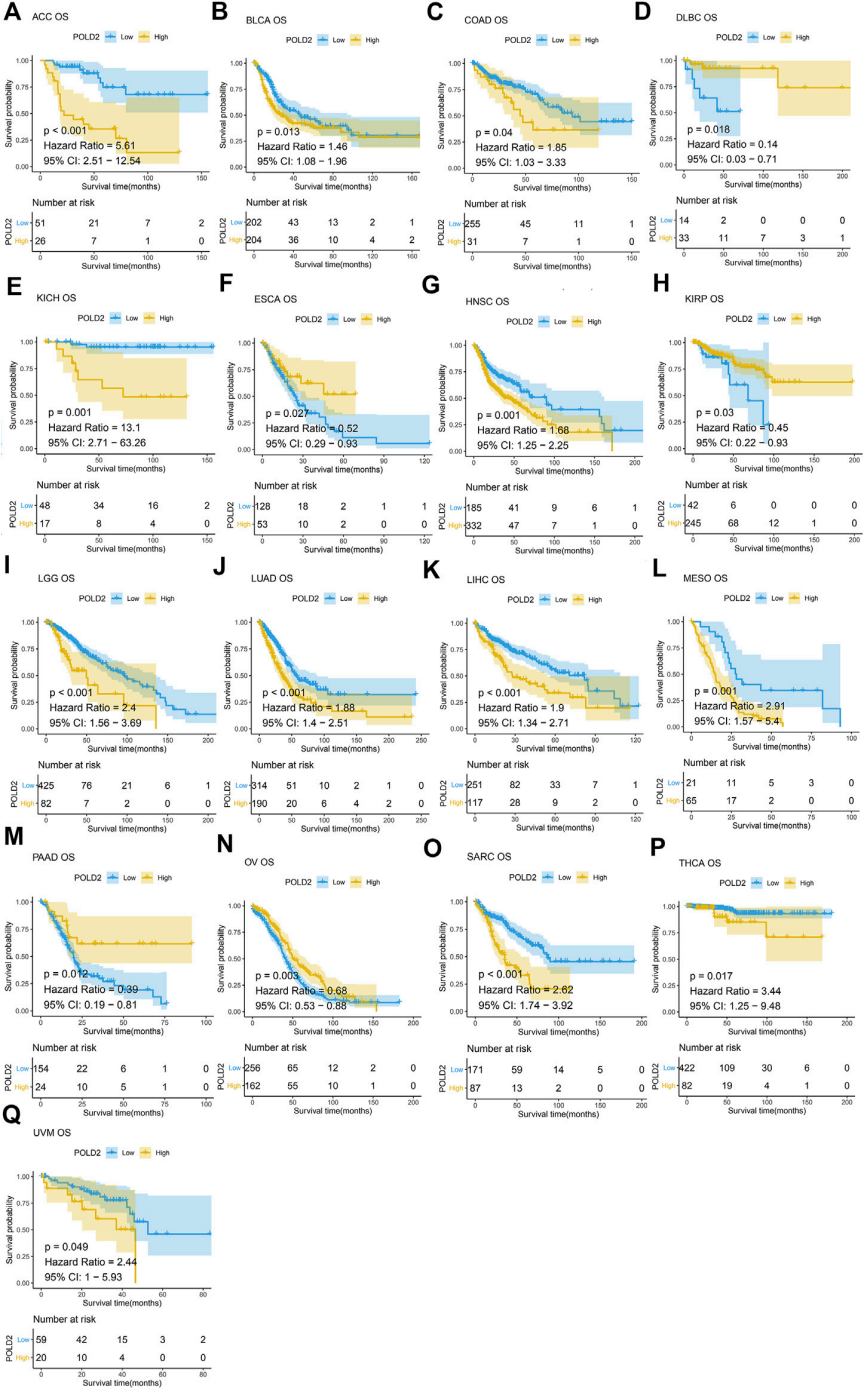
Kaplan-Meier analysis of OS correlation of POLD2. **(A–Q)** Survival curves of POLD2 in corresponding tumours.

**FIGURE 4:**
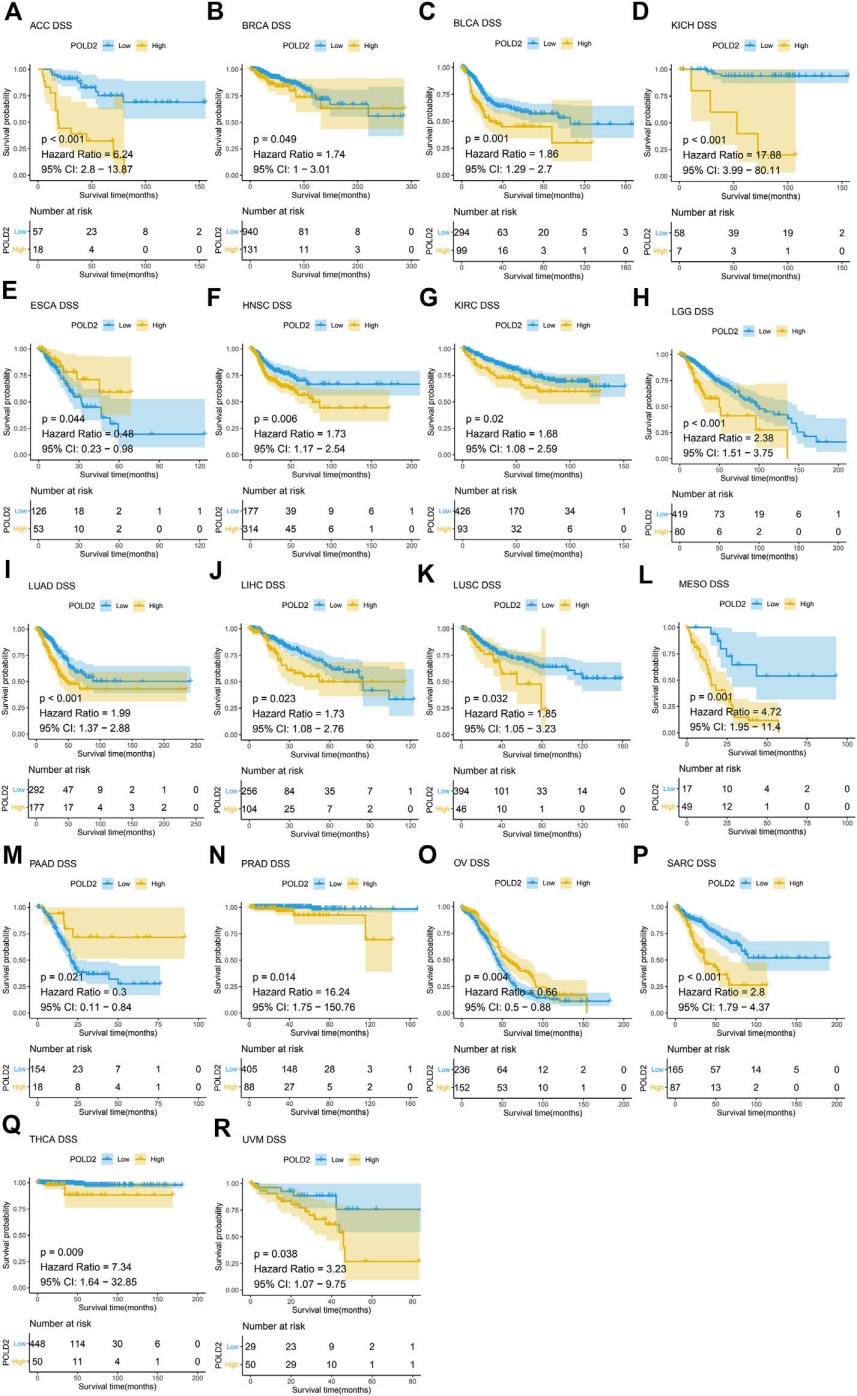
Kaplan-Meier analysis of DSS correlation of POLD2. **(A–R)** Survival curves of POLD2 in corresponding tumours.

**FIGURE 5:**
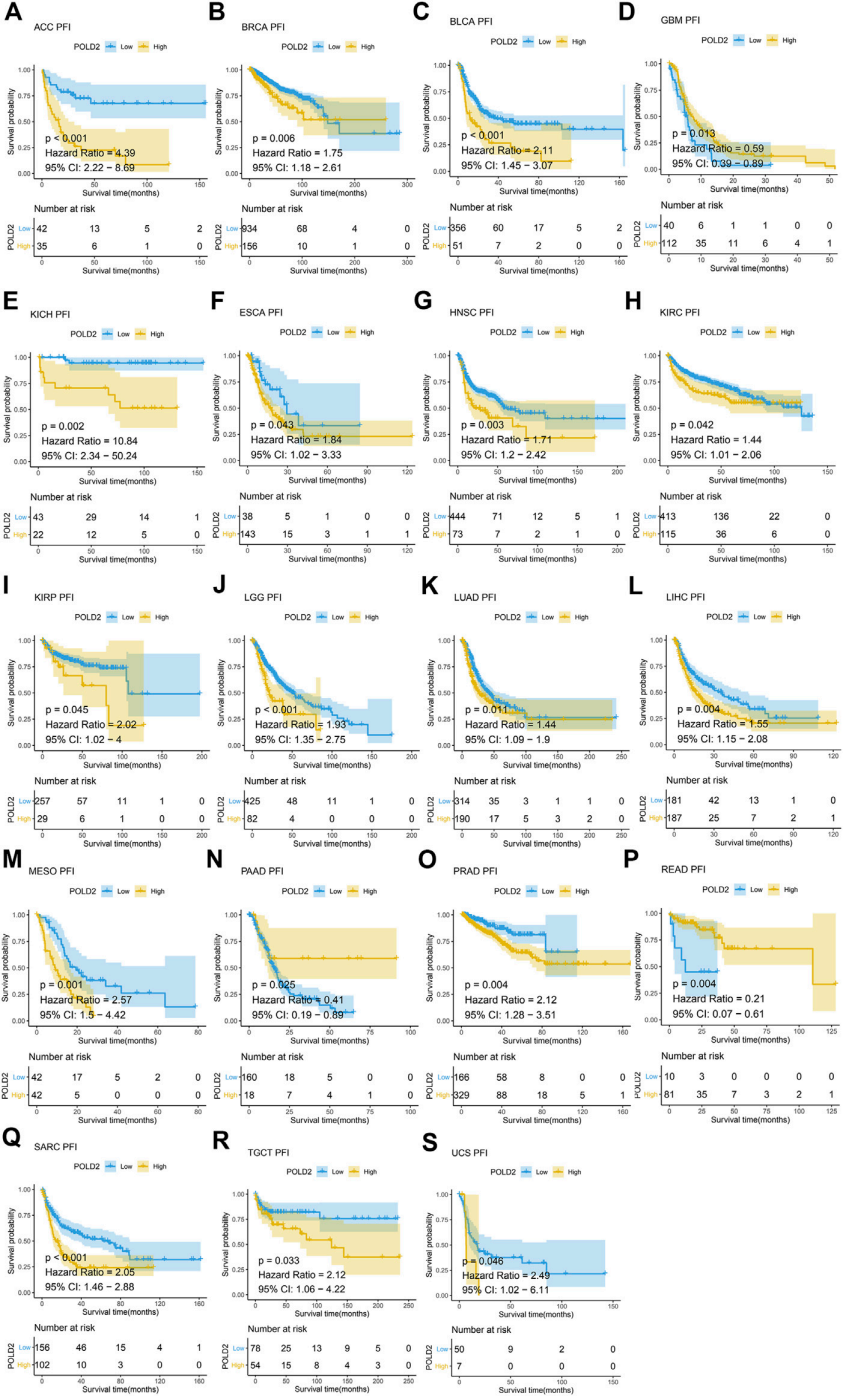
Kaplan-Meier analysis of PFI correlation of POLD2. **(A–S)** Survival curves of POLD2 in corresponding tumours.

**FIGURE 6:**
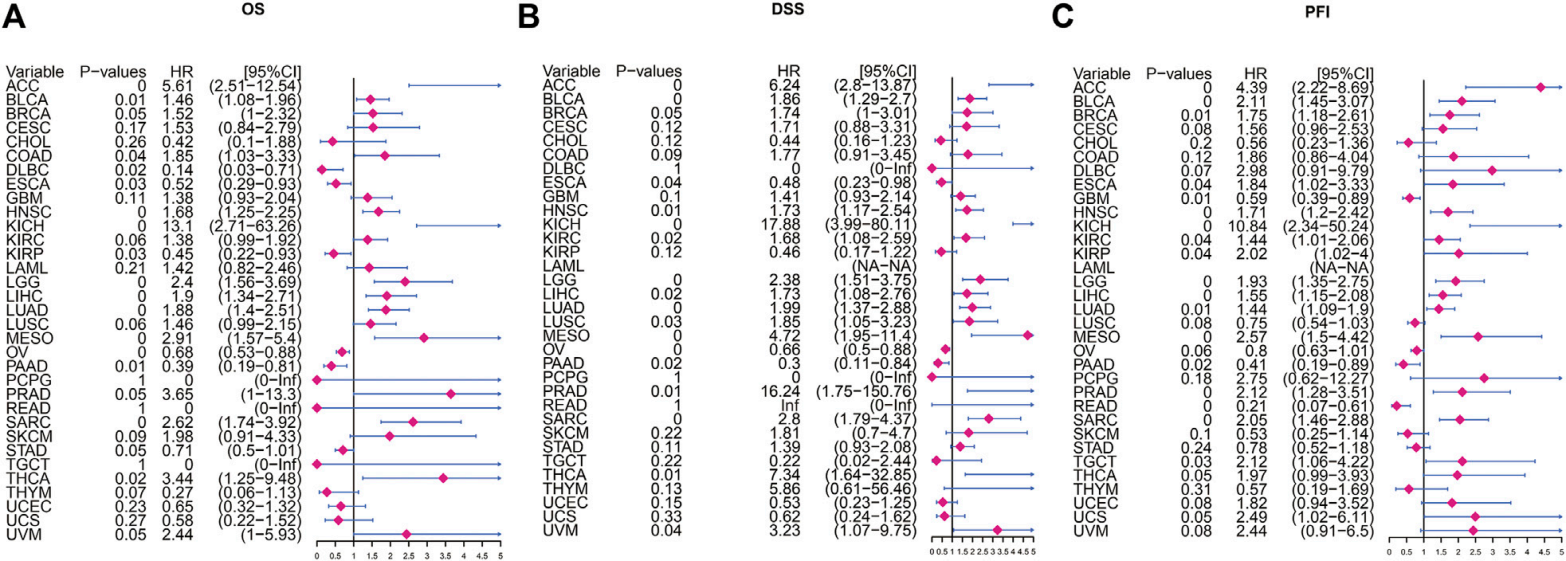
Univariate Cox regression analysis of POLD2. **(A)** Forest plot showing univariate Cox regression results of POLD2 on OS in TCGA pan-cancer. **(B)** Forest plot showing the univariate Cox regression results of POLD2 on DSS in TCGA pan-cancer. **(C)** Forest plot showing univariate Cox regression results of POLD2 on PFI in TCGA pan-cancer. Red color represents significant results.

Next, we use Kaplan-Meier survival curve analysis to evaluate the impact of POLD2 on the prognosis of different cancers. We used Kaplan-Meier to analyze the correlation between POLD2 and OS, DSS, and PFI in TCGA. We found that, especially in ACC, KICH, HNSC, LGG, MESO, and SARC (Figures 3–5), the expression level of POLD2 was negatively correlated with OS, DSS, and PFI (*p* < 0.05). These results indicate that the high expression of POLD2 may have a negative impact on the prognosis of cancer. In addition, through univariate COX analysis: OS results show that POLD2 is a risk factor for ACC, BLCA, COAD, HNSC, KICH, LGG, LIHC, LUAD, MESO, SARC, and THCA patients, and a protective factor for DLBC, ESCA, KIRP, OV, and PAAD patients (Figure 6A). DSS results show that POLD2 is a risk factor for patients with ACC, BLCA, HNSC, KICH, KIRC, LGG, LIHC, LUAD, LUSC, MESO, PRAD, SARC, THCA, UVM, and POLD2 is a protective factor for ESCA, OV, and PAAD patients (Figure 6B). PFI results show that POLD2 is a risk factor for patients with ACC, BLCA, BRCA, ESCA, HNSC, KICH, KIRC, KIRP, LGG, LIHC, LUAD, MESO, PRAD, SARC, and TGCT, and a protective factor for patients with GBM, PAAD, and READ (Figure 6C). It can be seen that high expression of POLD2 may lead to poor prognosis of patients.

### POLD2 expression and immune cells infiltration analysis

**FIGURE 7:**
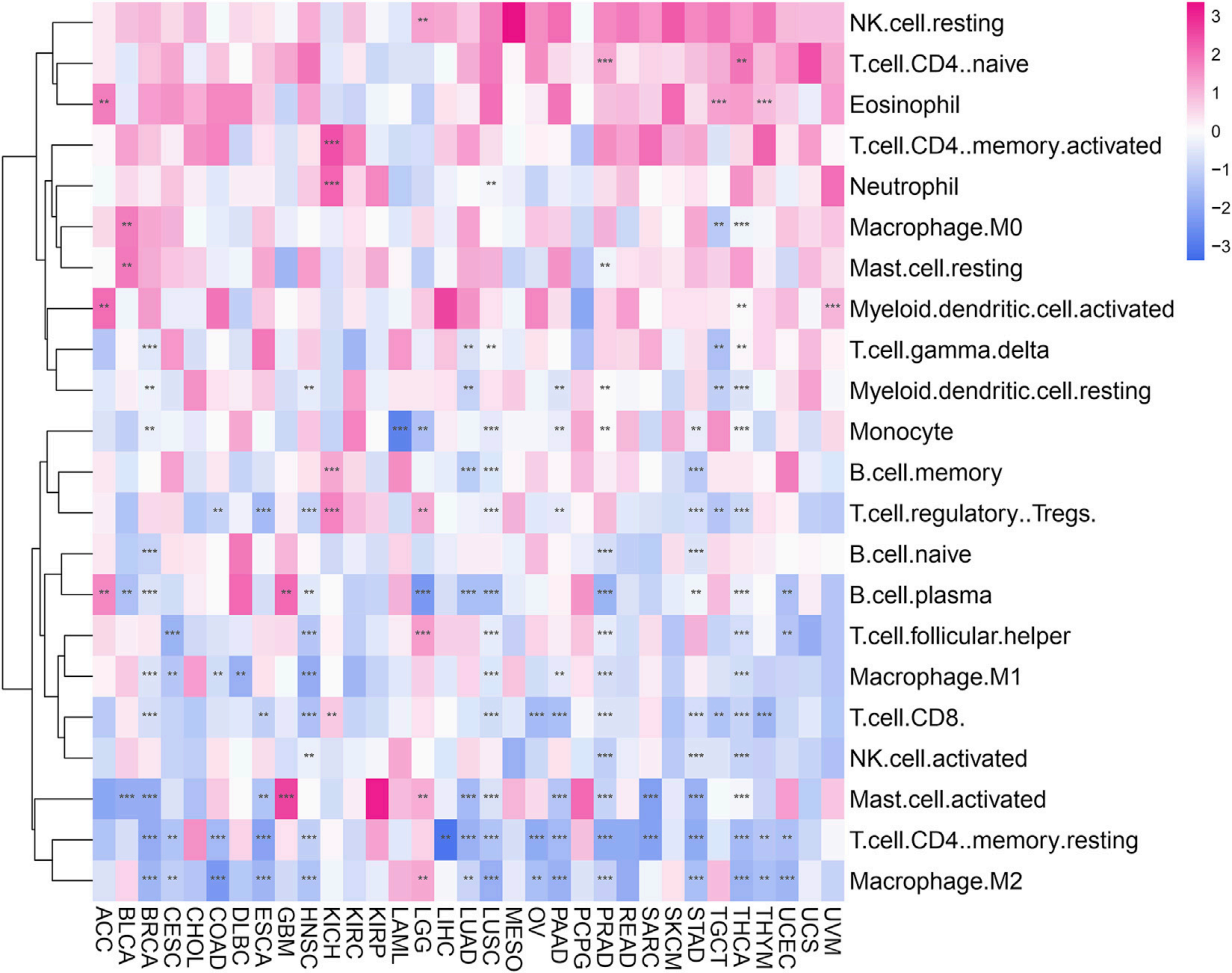
Immune cell infiltration analysis. Heat map shows the correlation of POLD2 expression in cancer with tumor immune infiltration.

We use the ESTIMATE algorithm to evaluate the expression of POLD2 in the tumor microenvironment through the TIMER database. It was found that in ACC, BRCA, CESC, COAD, DLBC, ESCA, GBM, HNSC, KICH, KIRC, LAML, LIHC, LUAD, LUSC, OV, PAAD, PRAD, SKCM, STAD, TGCT, THCA, UCEC, and POLD2 is negatively correlated with ImmuneScore (*p* < 0.05, Supplementary Figure S2). In ACC, BRCA, CESC, CHOL, COAD, DLBC, ESCA, GBM, HNSC, KIRC, KIRP, LAML, LIHC, LUAD, LUSC, MESO, OV, PAAD, PRAD, SARC, SKCM, STAD, TGCT, THCA, THYM, UCEC, and POLD2 is negatively correlated with StromalScore (*p* < 0.05, Supplementary Figure S3). In ACC, BRCA, CESC, COAD, DLBC, ESCA, GBM, HNSC, KICH, KIRC, KIRP, LAML, LIHC, LUAD, LUSC, OV, PAAD, PRAD, SARC, SKCM, STAD, TGCT, THCA, UCEC, and POLD2 is negatively correlated with ESTIMATEScore (*p* < 0.05, Supplementary Figure S4). It can be seen that POLD2 is negatively correlated with tumor purity. Next, we used the TIMER2 database to evaluate the correlation between POLD2 expression and tumor immune infiltration in cancer (Figure 7). The results showed that: Eosinophil was positively correlated with the expression of POLD2 in tumors (*p* < 0.05). T cell.CD8. Is negatively correlated with POLD2 expression in tumors (*p* < 0.05). T cell.CD4.memory.resting is negatively correlated with POLD2 expression in tumors (*p* < 0.05).

### The correlation between POLD2 and immune marker set tumor mutation burden and microsatellite instability

**FIGURE 8:**
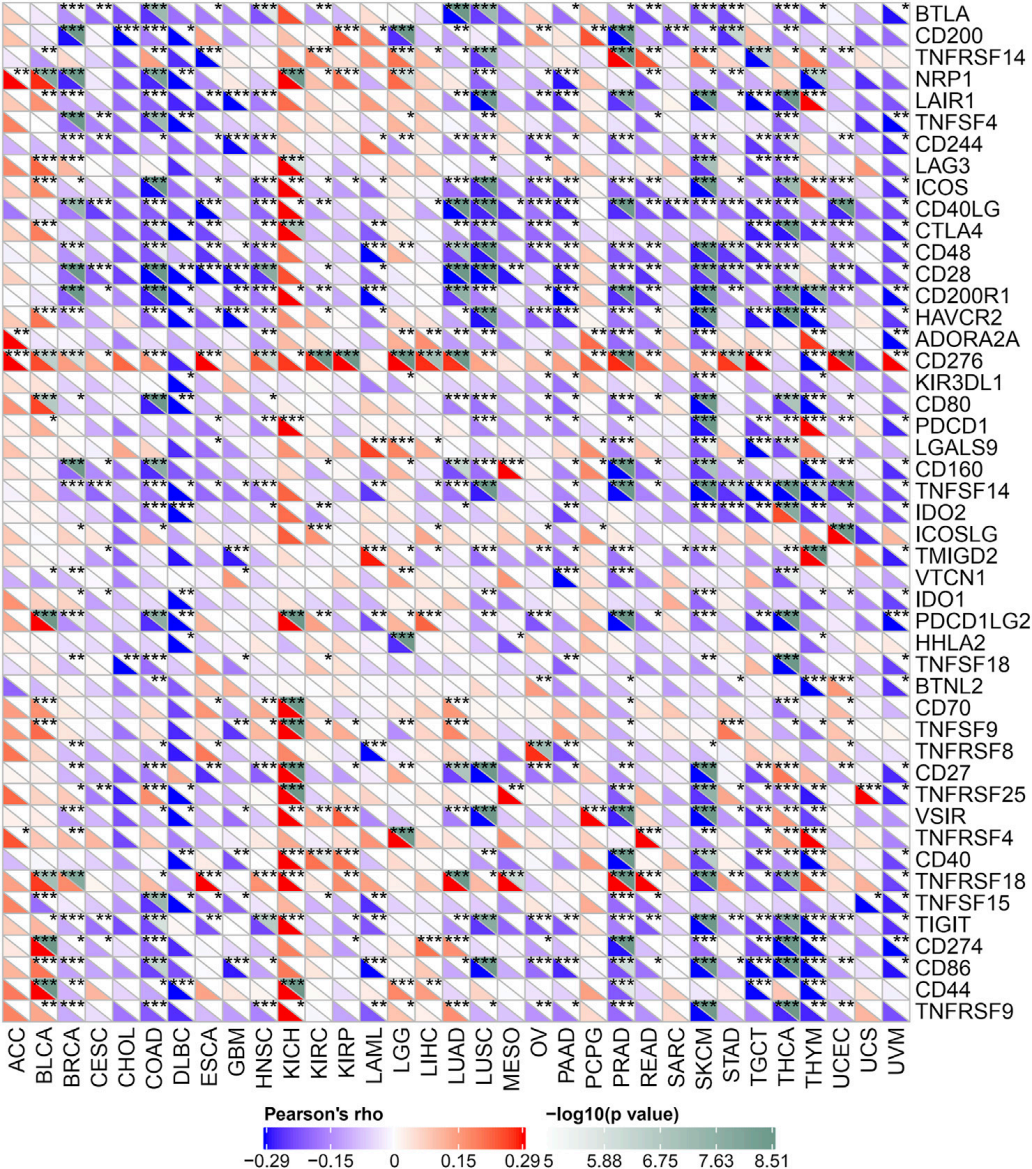
Relationship between POLD2 and immune checkpoint genes. Heatmap shows the relationship between immune checkpoint genes and POLD2 in different cancers.

**FIGURE 9:**
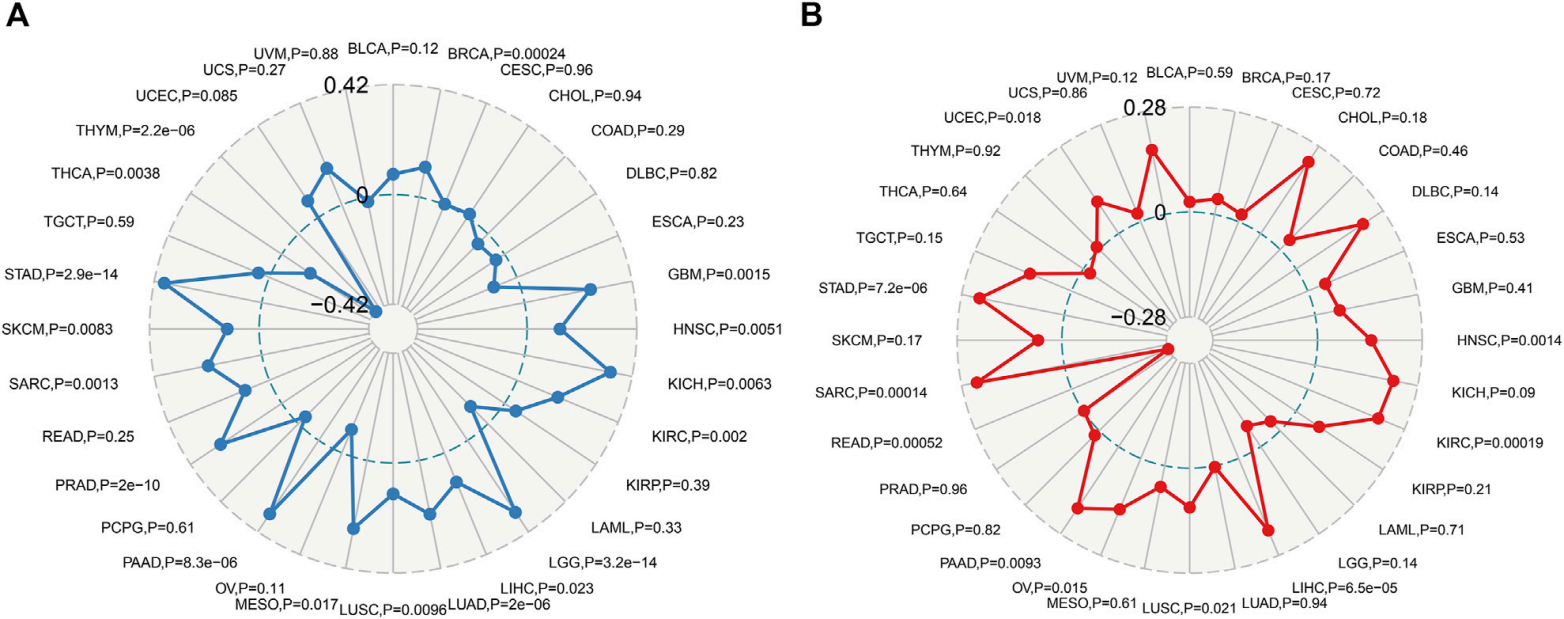
Correlation of POLD2 expression analysis with MSI and TMB. **(A)** Relationship between POLD2 expression and TMB in human cancers. **(B)** The relationship between POLD2 expression and MSI in human cancers. The Spearman’s correlation coefficient and *p*-value are shown in the radar plot.

Immune monitoring and immune escape are of great significance to the survival of cancer patients. Cancer cells can use immune checkpoint genes to evade immune surveillance. By examining the relationship between POLD2 and immune checkpoint genes (Figure 8), we found that in KICH, POLD2 expression is positively correlated with the expression of NRP1, LAG3, ICOS, CD40LG, CTLA4, CD200R1, CD276, PDCD1, PDCD1LG2, CD70, TNFSF9, CD27, TNFRSF25, VSIR, CD40, TNFRSF18, TIGIT, CD44, and TNFRSF9 (*p* < 0.05), this indicates that the high expression of POLD2 plays an important role in mediating immune evasion. After that, we used the expression of POLD2 in TCGA to analyze MSI and TMB, respectively (Figure 9). We found that POLD2 was negatively correlated with TMB in THCA and THYM(*p* < 0.05), while POLD2 was positively correlated with TMB in STAD, SKCM, SARC, PRAD, PAAD, MESO, LUSC, LUAD, LIHC, LGG, KIRC, KICH, HNSC, GBM, and BRCA (*p* < 0.05). And POLD2 is negatively correlated with MSI in SARC(*p* < 0.05), and POLD2 is positively correlated with MSI in UCEC, STAD, SARC, PAAD, OV, LUSC, LIHC, KIRC, and HNSC(*p* < 0.05). This indicates that POLD2 may be used as a new tumor marker.

### The correlation between POLD2 and immunoregulatory genes

**FIGURE 10:**
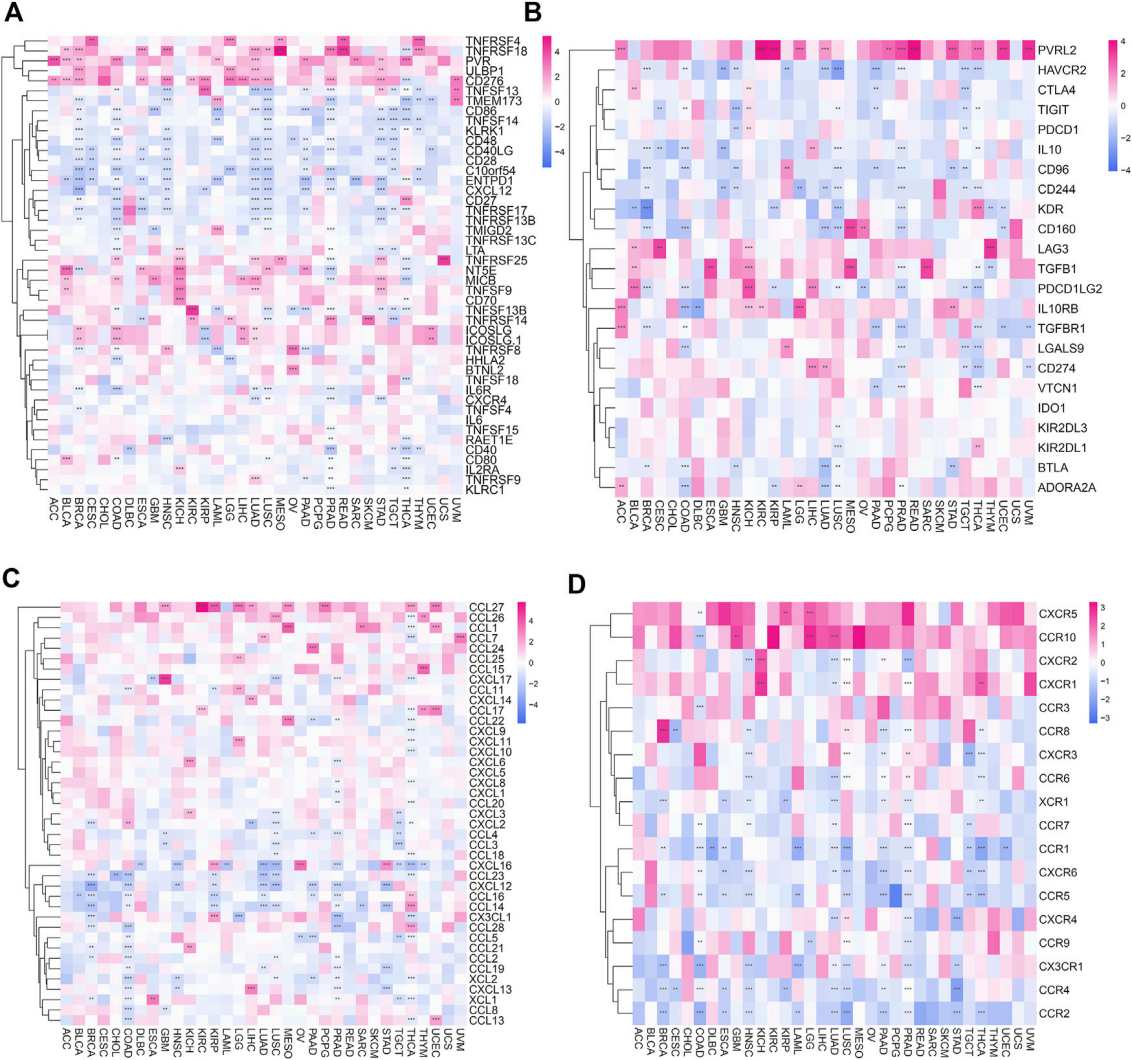
Correlation of POLD2 with immunomodulatory genes. **(A)** Correlation of immune activation genes in pan-cancer with POLD2. **(B)** Correlation of immunosuppressive genes in pan-cancer with POLD2. **(C)** Correlation of chemokines with POLD2 in pan-cancer. **(D)** Correlation of chemokine receptors with POLD2 in pan-cancer.

We researched the correlation of 46 immune activation genes with POLD2 in pan-cancer (Figure 10A). In most cancers, CD86, TNFSF14, KLRK1, CD48, CD40LG, CD28, C10orf54, ENTPD1, CXCL12, and POLD2 are negatively correlated (*p* < 0.05). In the analysis of 23 immunosuppressive genes (Figure 10B), PVRL2 was positively correlated with the expression of POLD2 in most cancers (*p* < 0.05). The results indicate that POLD2 may cause immunosuppression. In addition, we also studied the correlation between the expression of POLD2 and chemokines and their receptor genes (Figures 10C,D). The results show that in most cancers, the expression of chemokine CCL27 and POLD2 are positively correlated (*p* < 0.05). The expression of chemokines CCL4, CXCL16, CCL23, CXCL12, CCL16, and CCL14 was negatively correlated with the expression of POLD2 (*p* < 0.05). In most cancers, most chemokine receptors are negatively correlated with the expression of POLD2 (*p* < 0.05).

### MMRs gene expression

**FIGURE 11:**
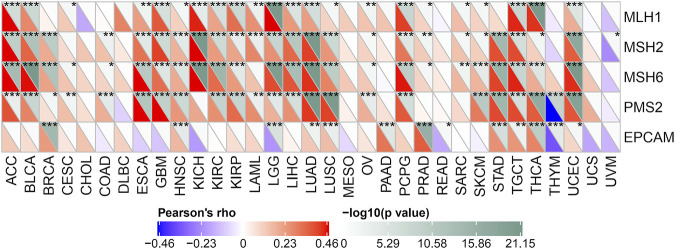
Correlation of MMRs genes with POLD2 expression. Heat map presents the relationship between MLH1, MSH2, MSH6, PMS2, and EPCAM mutations and POLD2 expression.

Here we use the expression profile data of TCGA to evaluate 5 MMRs genes: the relationship between MLH1, MSH2, MSH6, PMS2, and EPCAM mutations and the expression of POLD2 (Figure 11). We found that in most tumor samples, these 5 MMRs gene mutations were positively correlated with POLD2 expression (*p* < 0.05). This indicates that POLD2 may cause the loss of the function of key genes in the mismatch repair mechanism in the cell, resulting in DNA replication errors that cannot be repaired, leading to higher somatic mutations, and ultimately leading to the occurrence of tumors.

### Gene enrichment analysis and functional judgment

**FIGURE 12:**
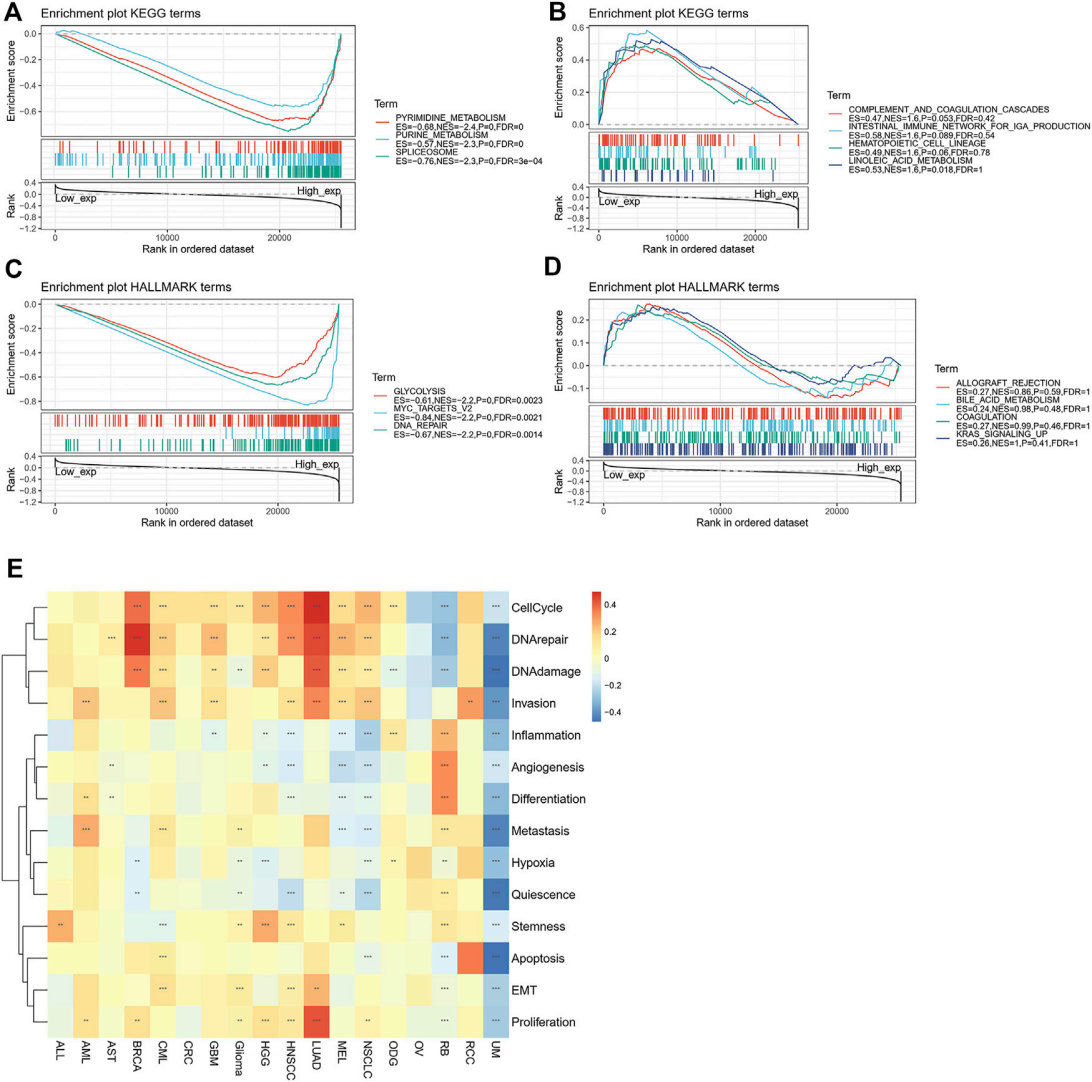
Enrichment analysis of POLD2-related genes. **(A)** Enrichment analysis of the POLD2 high expression group in the KEGG pathway. **(B)** Enrichment analysis of POLD2 low expression group in KEGG pathway. **(C)** Enrichment analysis of the POLD2 high expression group in the HALLMARK pathway. **(D)** Enrichment analysis of the POLD2 low expression group in the HALLMARK pathway. **(E)** Functional analysis of POLD2 at single cell level.

We used GSEA to identify the functional enrichment of high expression of POLD2 and low expression of POLD2 (Figure 12). The results showed that in the POLD2 high expression group, Pyrimidine Metabolism, Purine Metabolism, Spliceosome, Glycolysis, MYC_TARGETS_V2, and DNA_REPAIR expression increased (*p* < 0.05). It can be seen that the high expression of POLD2 is related to tumor occurrence and poor prognosis. We made a functional judgment on POLD2. The results showed that POLD2 was positively correlated with proliferation and cellcycle in LUAD (*p* < 0.05). POLD2 was positively correlated with DNArepair in BRCA (*p* < 0.05). POLD2 was negatively correlated with DNAdamage, quiescence and apoptosis in UM (*p* < 0.05).

### Association between POLD2 expression and immune checkpoint blockade response

**FIGURE 13:**
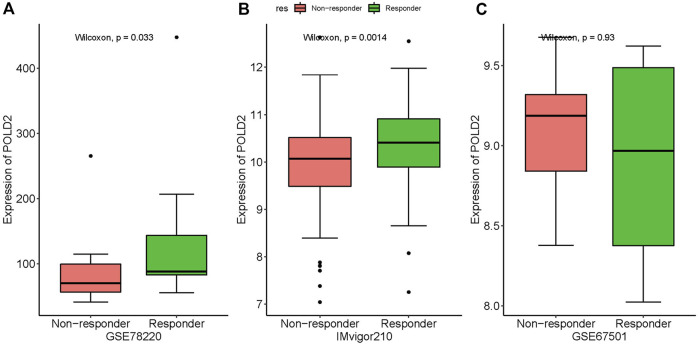
The association of POLD2 expression with immune checkpoint blockade (ICB) response. The association between POLD2 expression and ICB response. Data from the immunotherapy cohort GSE78220 **(A)**, IMvigor210 **(B)** and GSE67501 **(C)**, showing non-responder and responder.

The correlation between POLD2 expression and ICB response was validated by analyzing data from the immunotherapy cohort of uroepithelial carcinoma (IMvigor210). Significant differences were found in POLD2 expression in patients with different efficacies. POLD2 was highly expressed in patients with respond by analyzing the data from GSE78220 (*p* = 0.033, Figure 13A). Similarly, POLD2 expression was higher in patients with response than in those with not response in IMvigor210 (*p* = 0.0014, Figure 13B). However, we did not observe the above phenomenon in GSE67501 (*p* = 0.93, Figure 13C). It suggests that patients with high POLD2 expression could be a potentially beneficial population for ICB treatment.

### PPI network analysis

We extract 20 genes closely related to POLD2 expression for PPI network analysis. There are 20 points in the network, and there are 5 proteins interacting with POLD2 (Supplementary Figure S5).

## Discussion

POLD2 is a subunit of the DNA polymerase delta complex, which encodes a protein involved in DNA replication and repair (Perez et al., 2000). Many researches point out that the abnormal expression of POLD2 is frequently related to the occurrence, development or metastasis of human cancer. PLOD2, highly expressed in esophageal squamous cell carcinoma, promotes the invasion and metastasis of esophageal squamous cell carcinoma through the epithelial to mesenchymal transition *via* the FAK/AKT signal pathway (Di et al., 2019; Dongre and Weinberg, 2019). In addition, POLD2 is also associated with a poor prognosis in patients with bladder urothelial cancer (Elgaaen et al., 2010). Moreover, the upregulation of PLOD2 in osteosarcoma is related to lymphatic metastasis and distant metastasis. It may promote the invasion and metastasis of osteosarcoma through the FAK/JAK2-STAT3 signaling pathway (Cao et al., 2019). Despite the above findings, the functional role and mechanism of POLD2 in pan-cancer remains to be elucidated. The relationship between POLD2 overexpression and clinical parameters or prognosis needs to be further explored.

In this study, comparing the normal tissues, we analyzed the expression of POLD2 in various tumors in TGCA and the relationship between abnormal expression and prognosis. It was found that compared with the neighboring normal tissues, POLD2 was significantly overexpressed in a variety of tumors. In addition, survival analysis showed that the overexpression of POLD2 was poorly correlated with OS and DSS, while analyzing the survival curve of patients, it was found that the high expression of POLD2 was correlated with the lower survival rate of patients. And as the cancer stage progresses, the expression of POLD2 shows an upward trend in most cancers. These indicate that the high expression of POLD2 is associated with the poor prognosis of cancer patients, and it may result in a lower survival rate of patients.

In the normal body, the immune system performs an immune surveillance function, which can identify and eliminate abnormal cells in the body. Nonetheless, tumors may evade the immune system through different mechanisms, leading to further growth and invasion. For instance, cancer cells can use immune checkpoint genes such as PD-1 and CTLA-4 to evade immune surveillance. We examined the relationship between POLD2 expression and immune checkpoint genes, and then further analyzed the correlation with the degree of immune infiltration in human cancers. And it was found that high POLD2 expression plays an important role in mediating the immune escape of many tumors. MSI is a major predictive biomarker for the efficacy of immune checkpoint inhibitors (Cohen et al., 2019), and TMB can be regarded as a marker for the treatment response of immune checkpoint inhibitors (Chan et al., 2019). We evaluated MSI and TMB and it showed that high POLD2 expression was positively correlated with MSI, and negatively correlated with TMB in a variety of tumors. MSI is associated with a higher risk of cancer and with clinicopathological features, such as higher quality of tumor infiltrating lymphocytes (Cohen et al., 2019). Therefore, our work clarifies the potential role of POLD2 in cancer immunology and its prognostic value to human cancer.

Furthermore, POLD2 plays a vital role in the occurrence and development of tumors. In our study, pathway enrichment analysis demonstrated that the high expression of POLD2 is associated with abnormal pyrimidine and purine metabolism, which play a vital role in the occurrence and development of cancer in the early stage (Yin et al., 2018). At the same time, we noticed that the high expression of POLD2 is also related to MYC targets v2, and the high MYC target score is related to tumor aggressiveness and poor prognosis in metastatic adenocarcinoma (Schulze et al., 2020). In addition, we found that the high expression of POLD2 is negatively correlated with some immune-activating genes, and positively correlated with some immunosuppressive genes in a variety of tumors, which may inhibit the tumor suppressor effect of related immune cells (Takenaka et al., 2019; Whelan et al., 2019), thereby helping the immune evasion of the tumor. This may indicate the potential of POLD2 as an immune checkpoint.

Although having explored and integrated information from various databases, we acknowledge that our research has certain limitations. First of all, though we have obtained some meaningful insights into POLD2 in human cancer from data mining, it will be beneficial to clinical applicability if these findings are verified by cell or animal experiments. In addition, more clinical trials are needed to verify the immune checkpoint function of POLD2.

In summary, the results of the present study clarify the strong correlation between POLD2 expression and the prognosis of various cancers. Since POLD2 is overexpressed in a variety of human cancers and is associated with worse survival, it may serve as a potential target for cancer management. Furthermore, our research provides insights into the underlying mechanism by which POLD2 overexpression may regulate tumor immunity. In the future, more basic and clinical researches based on POLD2 and tumor microenvironment may help us to optimize the clinical strategy of cancer treatment.

## Conclusion

In this study, a pan-cancer analysis of POLD2 was performed, and the results showed that the upregulation of POLD2 expression is associated with the poor prognosis of many tumors. Furthermore, POLD2 is also involved in tumor immune escape, which may be used as an immune checkpoint for cancer treatment. Our research showed that POLD2 is a potential prognostic biomarker, which can be used for clinical tumor prognosis prediction and immunotherapy assessment.

## Data Availability

The original contributions presented in the study are included in the article/Supplementary Material, further inquiries can be directed to the corresponding authors.
