# Supplementary material for: An integrative analysis revealing POLD2 as a tumor suppressive immune protein and prognostic biomarker in pan-cancer
Source: Front Genet. 2022 Aug 23;13:877468. doi: 10.3389/fgene.2022.877468 (PMC9447486; doi:10.3389/fgene.2022.877468)
Supplement: Supplementary file 1 [file DataSheet1.docx]

Supplementary Material

## Supplementary Figures


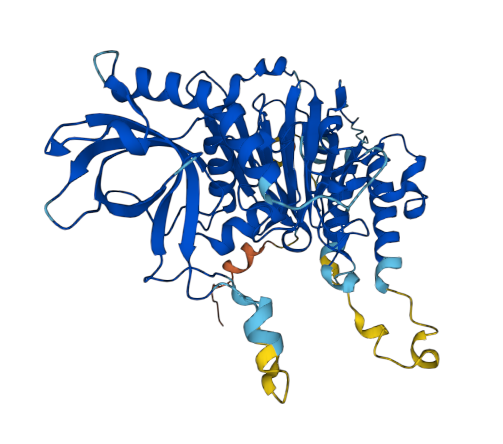


**Figure S1.** Structure analysis of POLD2.


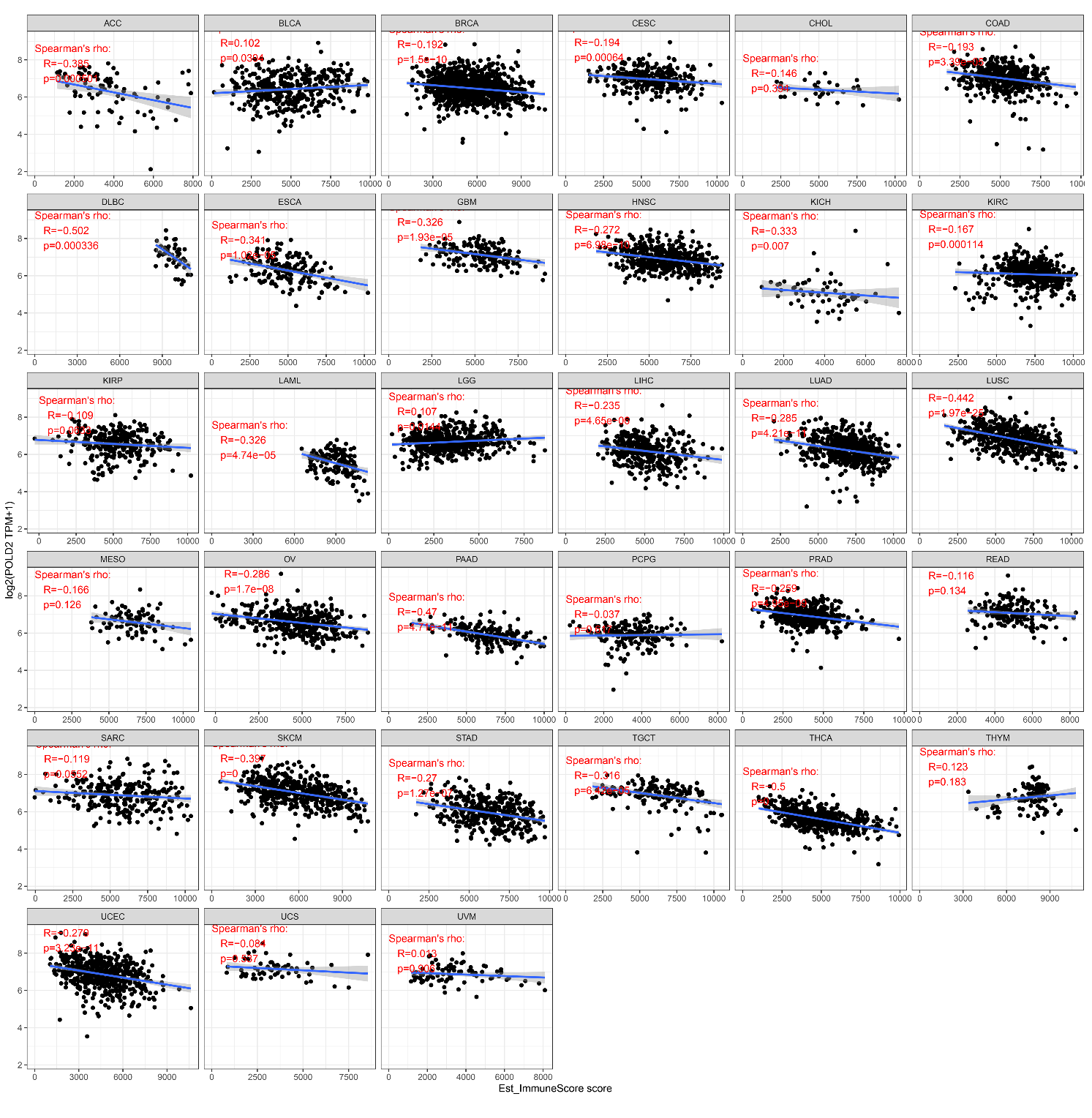


**Figure S2**. Analysis of tumor Immunescore.


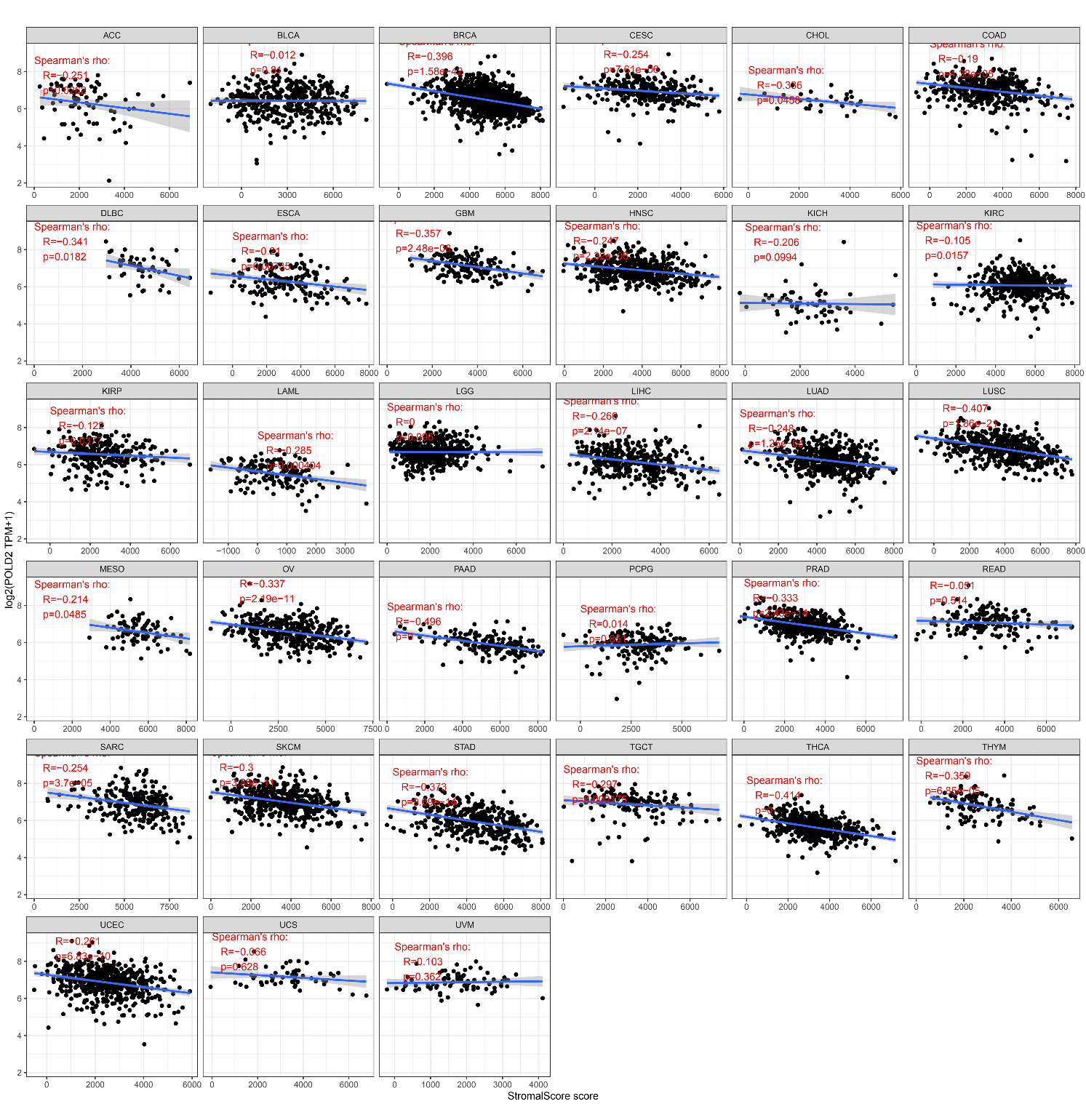


**Figure S3.** Analysis of tumor StromalScore.


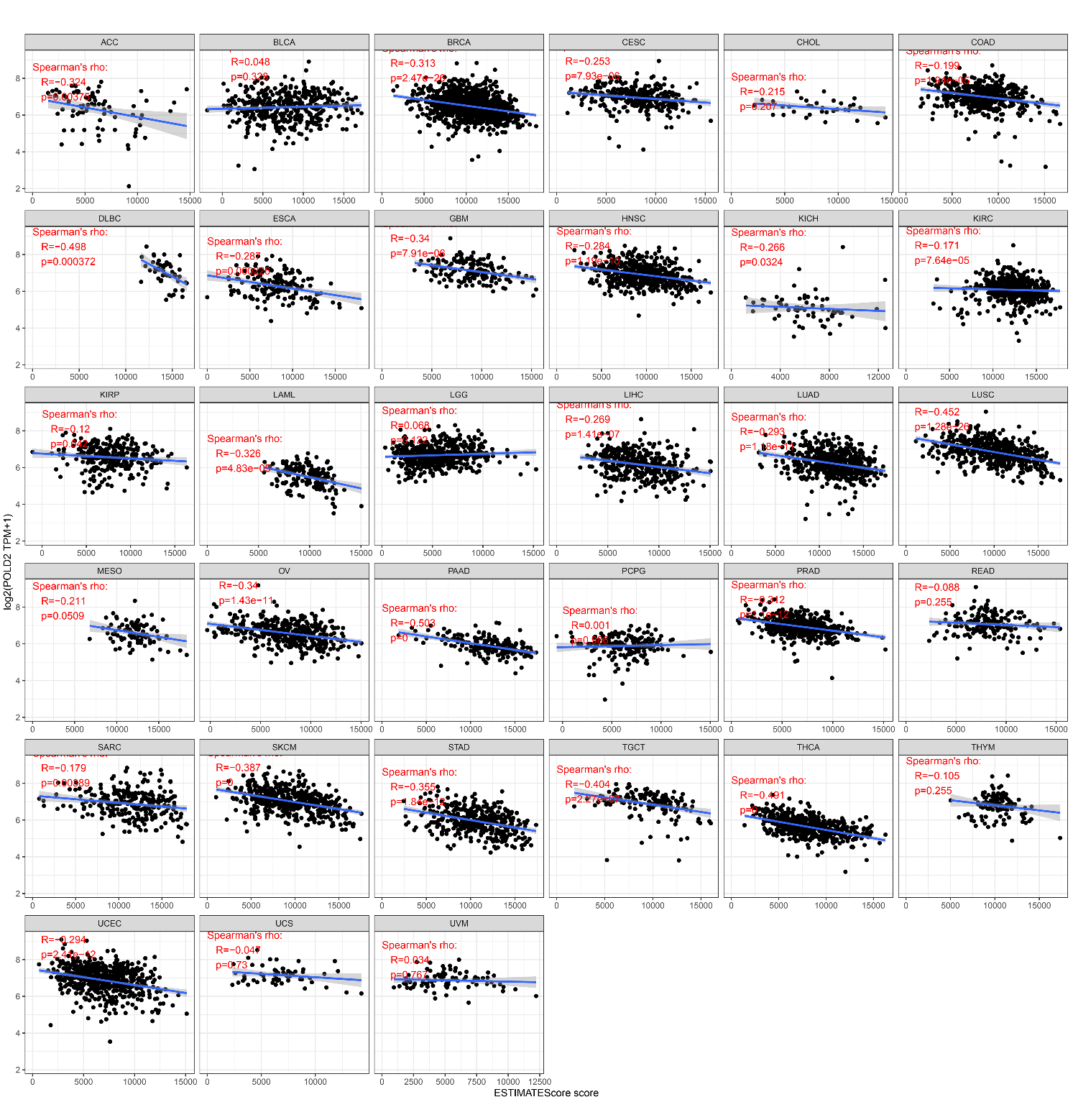


**Figure S4.** Analysis of tumor ESTIMATEScore.


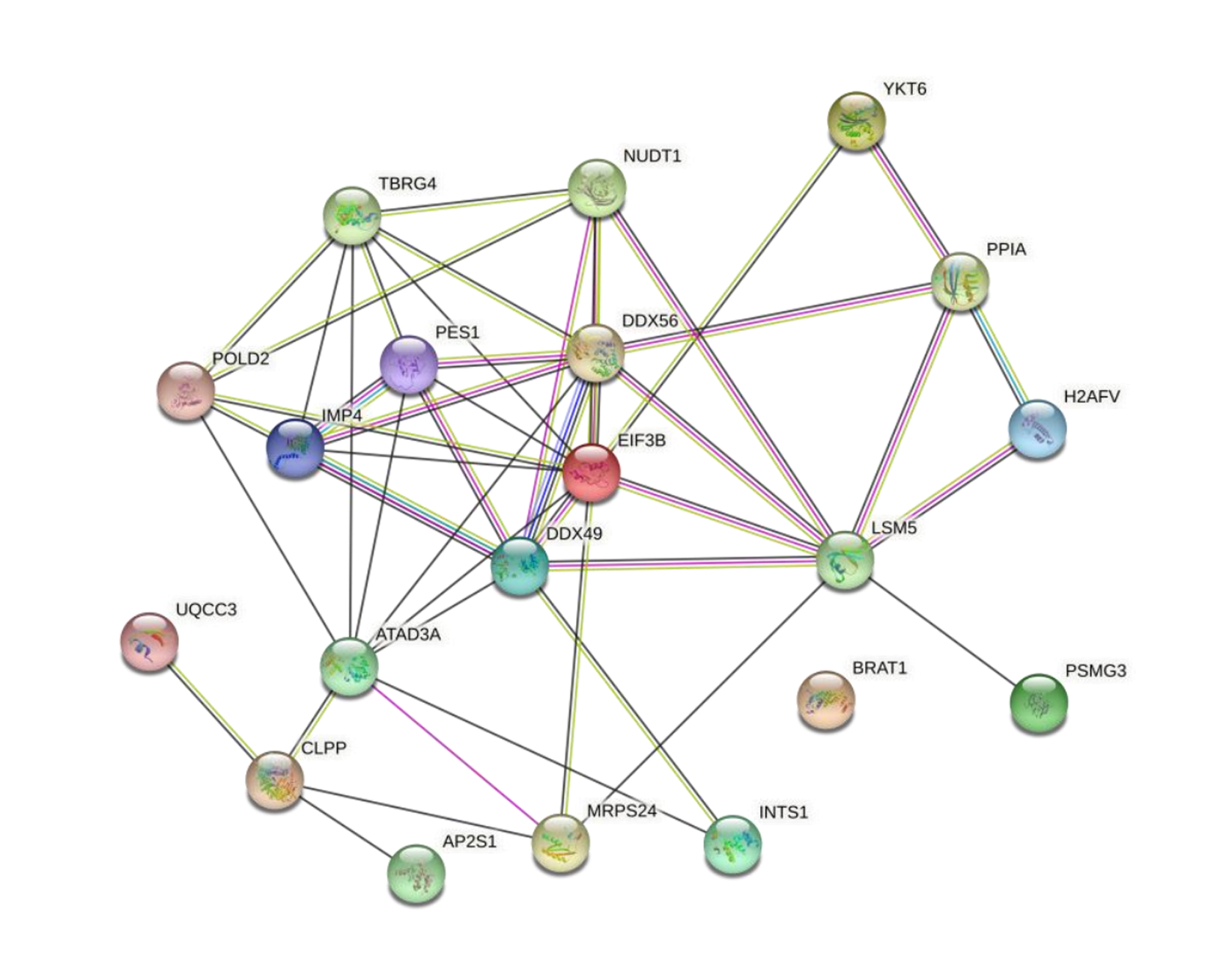


**Figure S5**. PPI Network Analysis.
